# Association of Accelerometer-Measured Sedentary Time and Physical Activity With Risk of Stroke Among US Adults

**DOI:** 10.1001/jamanetworkopen.2022.15385

**Published:** 2022-06-03

**Authors:** Steven P. Hooker, Keith M. Diaz, Steven N. Blair, Natalie Colabianchi, Brent Hutto, Michelle N. McDonnell, John E. Vena, Virginia J. Howard

**Affiliations:** 1 College of Health and Human Services, San Diego State University, San Diego, California; 2 Department of Medicine, Columbia University Irving Medical Center, New York, New York; 3 Department of Exercise Science, Arnold School of Public Health, University of South Carolina, Columbia; 4 Department of Epidemiology and Biostatistics, Arnold School of Public Health, University of South Carolina, Columbia; 5 School of Kinesiology, University of Michigan, Ann Arbor; 6 Prevention Research Center, Arnold School of Public Health, University of South Carolina, Columbia; 7 SA Health, Northern Adelaide Local Health Network, Modbury, South Australia; 8 Department of Public Health Sciences, Medical University of South Carolina, Charleston; 9 Department of Epidemiology, School of Public Health, University of Alabama at Birmingham, Birmingham

## Abstract

**Importance:**

The amount and intensity of physical activity required to prevent stroke are yet to be fully determined because of previous reliance on self-reporting measures. Furthermore, the association between objectively measured time spent being sedentary as an independent risk factor for stroke is unknown.

**Objective:**

To investigate the associations of accelerometer-measured sedentary time and physical activity of varying intensity and duration with the risk of incident stroke.

**Design, Setting, and Participants:**

This cohort study involved participants who were enrolled in the Reasons for Geographic and Racial Differences in Stroke (REGARDS) study from February 5, 2003, to October 30, 2007. Accelerometer data were collected from 7607 Black and White adults 45 years or older in the contiguous US between May 12, 2009, and January 5, 2013. Data on other races and ethnicities were not collected for scientific and clinical reasons. By design, Black adults and residents of the southeastern US stroke belt and stroke buckle were oversampled. Data were analyzed from May 5, 2020, to November 11, 2021.

**Exposures:**

Sedentary time, light-intensity physical activity (LIPA), and moderate- to vigorous-intensity physical activity (MVPA) were measured using a hip-mounted accelerometer worn for 7 consecutive days and stratified by tertile for the analyses.

**Main Outcomes and Measures:**

Incident stroke.

**Results:**

Among 7607 participants, the mean (SD) age was 63.4 (8.5) years; 4145 participants (54.5%) were female, 2407 (31.6%) were Black, and 5200 (68.4%) were White. A total of 2523 participants (33.2%) resided in the stroke belt, and 1638 (21.5%) resided in the stroke buckle. Over a mean (SD) of 7.4 (2.5) years of follow-up, 286 incident stroke cases (244 ischemic [85.3%]) occurred. The fully adjusted hazard ratios (HRs) for incident stroke in the highest tertile compared with the lowest tertile were 0.74 (95% CI, 0.53-1.04; *P* = .08) for LIPA and 0.57 (95% CI, 0.38-0.84; *P* = .004) for MVPA. Higher sedentary time was associated with a 44% greater risk of incident stroke (HR, 1.44; 95% CI, 0.99-2.07; *P* = .04). When comparing the highest with the lowest tertile, mean sedentary bout duration was associated with a significantly greater risk of incident stroke (HR, 1.53; 95% CI, 1.10-2.12; *P* = .008). After adjustment for sedentary time, the highest tertile of unbouted MVPA (shorter bouts [1-9 minutes]) was associated with a significantly lower risk of incident stroke compared with the lowest tertile (HR, 0.62; 95% CI, 0.41-0.94; *P* = .02); however, bouted MVPA (longer bouts [at least 10 minutes]) was not (HR, 0.78; 95% CI, 0.53-1.15; *P* = .17). When expressed as continuous variables, sedentary time was positively associated with incident stroke risk (HR per 1-hour/day increase in sedentary time: 1.14; 95% CI, 1.02-1.28; *P* = .02), and LIPA was negatively associated with incident stroke risk (HR per 1-hour/day increase in LIPA: 0.86; 95% CI, 0.77-0.97; *P* = .02).

**Conclusions and Relevance:**

In this cohort study, objectively measured LIPA, MVPA, and sedentary time were significantly and independently associated with incident stroke risk. Longer sedentary bout duration was also independently associated with an increased risk of incident stroke. These findings suggest that replacing sedentary time with LIPA, or even very short bouts of MVPA, may lower stroke risk, supporting the concept of moving more and sitting less as a beneficial stroke risk reduction strategy among adults.

## Key Points

**Question:**

Are physical activity of varying intensity and duration and time spent in sedentary behavior associated with risk of incident stroke in middle-aged and older US adults?

**Findings:**

In this cohort study of 7607 adults, greater accumulation of light-intensity and moderate- to vigorous-intensity physical activity were both associated with a reduced risk of stroke. Greater time spent being sedentary and longer bouts of sedentary time were associated with a higher risk of stroke.

**Meaning:**

This study’s findings suggest that more time spent being physically active, especially at moderate intensities, and less time spent being sedentary, particularly in longer bouts, may help reduce the risk of stroke.

## Teaser

This cohort study assesses the associations of accelerometer-measured sedentary time and physical activity of varying intensity and duration with the risk of incident stroke among adults 45 years or older in the US.

## Introduction

Stroke is currently the second most common cause of death and the third most common cause of disability globally,^1^ translating to approximately 6.5 million stroke deaths, 113 million disability-adjusted life-years, and 10.3 million new strokes worldwide. In the US alone, 795 000 people experience a stroke each year, which equates to 1 person every 40 seconds; on average, someone in the US dies of a stroke every 4 minutes.^2^

Stroke has been well established as a preventable disease^3^ that is associated with modifiable cardiovascular risk factors, such as hypertension, diabetes, dyslipidemia, obesity, tobacco use, and physical inactivity.^3,4^ Independent of other risk factors, physical activity has emerged as a significant factor associated with risk of stroke incidence.^3^ However, the amount and intensity of physical activity required to prevent stroke is yet to be fully determined because studies have relied on self-reporting measures that are subject to recall bias and overestimation of total physical activity.^5^ Furthermore, the association between sedentary behavior as an independent risk factor and stroke is unknown. Thus, the purpose of this study was to investigate the associations of accelerometer-measured sedentary time and physical activity of varying intensity and duration with the risk of stroke in a national cohort of middle-aged and older women and men. We hypothesized that less time spent being physically active and more time spent being sedentary would be associated with an increased risk of incident stroke in middle-aged and older adults.

## Methods

### Study Population

Participants were enrolled in the Reasons for Geographic and Racial Differences in Stroke (REGARDS) study; the design and methods of the REGARDS study are described elsewhere.^6^ Because the non-Hispanic Black adult population has the highest stroke mortality in the US compared with all other racial groups,^6^ the study was restricted to non-Hispanic Black and White individuals, with the White individuals serving as the reference group. There are also substantial geographic disparities in stroke mortality.^6^ A total of 30 239 Black and White participants 45 years or older from the contiguous US were enrolled from February 5, 2003, to October 30, 2007, using a combination of mail and telephone recruitment. By design, Black adults and residents of the southeastern US stroke belt (a region characterized by a high stroke mortality rate, which includes Alabama, Arkansas, Georgia, Louisiana, Mississippi, North Carolina, South Carolina, and Tennessee) and stroke buckle (a region within the larger stroke belt that is characterized by an even higher stroke mortality rate and comprises the coastal plain region of Georgia, North Carolina, and South Carolina) were oversampled to increase statistical power for assessing differences in disparities. At baseline, data on demographic characteristics, medications used, and cardiovascular risk factors were collected through a combination of telephone interviews and in-home physical examinations. The examinations included measurement of height, weight, blood pressure, blood and urine samples, and electrocardiography.

Participants were followed up at 6-month intervals to ascertain vital status (eg, general health status, hospitalizations, and stroke symptoms). Using an accelerometer, objective measures of physical activity and sedentary behavior were collected from REGARDS participants from May 12, 2009, to January 5, 2013 (mean [SD] time from enrollment, 5.7 [1.5] years; range, 1.9-9.5 years).^7^ The analytic sample in the current cohort study comprised 7607 participants who adhered to accelerometer wear requirements (≥4 days with accelerometer wear ≥10 hours), provided follow-up data, and did not have a self-reported history of stroke at baseline or adjudicated incident stroke before wearing the accelerometer (eFigure 1 in the Supplement). Data were analyzed from May 5, 2020, to November 11, 2021.

Characteristics of participants who agreed vs declined to wear the accelerometer and participants who were adherent vs nonadherent to accelerometer wear have been reported elsewhere.^7,8^ The study protocol was approved by institutional review boards at all participating institutions, and participants provided written informed consent for the parent REGARDS study and verbal informed consent for the current accelerometer cohort study. This study followed the Strengthening the Reporting of Observational Studies in Epidemiology (STROBE) reporting guideline for cohort studies.

### Accelerometer Data

Detailed methods used for accelerometer data collection and management have been described elsewhere.^7^ In brief, participants were provided with an accelerometer (Actical; Philips Respironics) to secure to their right hip and instructed to wear the device during waking hours for 7 consecutive days. The accelerometer has been validated for measurement of physical activity and sedentary behavior and reported to have acceptable reliability.^9,10^

Activity counts were summed over 1-minute epochs. Nonwear periods were defined as more than 150 consecutive minutes of 0 activity counts. This nonwear algorithm was previously validated (94% sensitivity and 73% specificity) against daily log sheets among REGARDS participants.^11^ Sedentary behavior was defined as 0 to 49 activity counts per minute, light-intensity physical activity (LIPA) as 50 to 1064 activity counts per minute, and moderate- to vigorous-intensity physical activity (MVPA) as 1065 activity counts or more per minute.^12^ A sedentary bout was defined as consecutive minutes in which the accelerometer registered fewer than 50 counts per minute. A bout of physical activity was defined as consecutive minutes in which the accelerometer registered 50 counts per minute or more. Sedentary behavior and physical activity variables were summed across each adherent day (defined as ≥10 hours of wear); means were then calculated across all of a participant’s adherent days to derive values per day.

Because of a high correlation between total sedentary time and wear time, the impact of variation in wear time was corrected by standardizing total sedentary time to 16 hours of wear time per day using the residuals obtained when regressing total sedentary time on wear time.^13^ First, we regressed measured total sedentary time against accelerometer wear time and calculated residuals to represent the observed minus estimated sedentary time. We then summed each participant’s residual sedentary value with the mean estimated sedentary time based on a wear time of 16 hours per day. As a result, total sedentary time was reported as the estimated minutes per day of sedentary time based on a standardized 16 hours of accelerometer wear. Participant characteristics stratified by level of wear time are shown in eTable 1 in the Supplement.

### Stroke Ascertainment

Event ascertainment methods used in the REGARDS study have been published previously.^14^ Participants (or their proxies) were contacted at 6-month intervals to ascertain potential stroke events. Deaths and causes of death were ascertained. Medical records were obtained for suspected stroke events and reviewed by separate adjudication committees. Stroke events were defined based on the World Health Organization definition as “rapidly developing clinical signs of focal, at times global, disturbance of cerebral function, lasting more than 24 hours or leading to death with no apparent cause other than that of vascular origin”^15^ or by review of final neuroimaging reports consistent with acute ischemia. Strokes were classified as ischemic or hemorrhagic and combined for this analysis. For this analysis, strokes identified after the last date of accelerometer wear through March 31, 2020, were included.

### Statistical Analysis

Participants were separately stratified into tertiles according to sedentary time (tertile 1: <11.8 hours/16-hour day; tertile 2: ≥11.8 to <13.0 hours/16-hour day; tertile 3: ≥13 hours/16-hour day), LIPA (tertile 1: <154.0 minutes/day; tertile 2: ≥154.0 to <220.4 minutes/day; tertile 3: ≥220.4 minutes/day), and MVPA (tertile 1: <2.7 minutes/day; tertile 2: ≥2.7 to <14.0 minutes/day; tertile 3: ≥14.0 minutes/day). Competing risks Cox proportional hazards regression modeling was used to calculate the hazard ratio (HR) for incident stroke associated with tertiles of LIPA, MVPA, and sedentary time, with death as the competing risk and treating age as the time scale. Unadjusted HRs were initially calculated and were then adjusted for age (<65 years or ≥65 years), race (Black or White), sex (male or female), region of residence (stroke belt, stroke buckle, or non–stroke belt/non–stroke buckle), educational level (less than high school, high school graduate, some college, or college graduate), and season in which the accelerometer was worn (summer, autumn, winter, or spring) in model 1. Model 2 was further adjusted for current smoking, alcohol consumption, atrial fibrillation, left ventricular hypertrophy, and history of coronary heart disease (CHD). Additional adjustment for MVPA (for sedentary time and LIPA) or sedentary time (for MVPA), expressed continuously, was performed in model 3.

Tests for linear trend across tertiles were conducted by including the tertile for each participant as an ordinal variable in regression models. Analyses were then repeated in a fully adjusted model that tested interactions for age (<65 years and ≥65 years), sex (male and female), race (Black and White), body mass index (BMI; calculated as weight in kilograms divided by height in meters squared) category (normal weight [BMI of 18.5-24.9] and overweight [BMI of 25.0-29.9] or obesity [BMI of 30 or higher]), and whether the participant did or did not meet physical activity guidelines (≥150 minutes/week of MVPA and <150 minutes/week of MVPA). Proportional hazards assumptions were confirmed with a Kolmogorov-type supremum test. Because region of residence violated the proportional hazards assumption, this variable was included as a cross-product with follow-up time in all models.

As secondary analyses, we also adjusted for risk factors (ie, body mass index, diabetes, and hypertension) that may have mediated the association between physical activity and stroke risk. Primary models did not include adjustment for these covariates because inclusion of intermediary variables can introduce overadjustment bias.^16^ We also examined the continuous dose-response association of LIPA, MVPA, and sedentary time with incident stroke in a fully adjusted model (model 3). We examined possible nonlinear associations nonparametrically with restricted cubic splines.^17^ Tests for nonlinearity used the likelihood ratio test, comparing the model with only the linear term vs the model with the linear and cubic spline terms. No nonlinear associations were identified for LIPA and sedentary time; thus, linear models were used for these measures. For MVPA, cubic polynomials were fitted with 0 minutes per day set as the reference, and restrictions were placed on the resulting curve to ensure a smooth appearance using 3 knots placed at the 5th, 20th, and 70th percentiles.

The accumulation of sedentary time in prolonged, uninterrupted bouts has received considerable interest as potentially the most hazardous form of sedentary behavior. Moreover, it has been suggested that there might be health benefit to short episodes of physical activity (contrary to previous guidelines endorsing bouts of ≥10 minutes).^18^ Thus, as a tertiary analysis, we examined the association of mean sedentary bout duration (a measure of overall prolonged, uninterrupted sedentary behavior; tertile 1: <8.3 minutes/bout; tertile 2: ≥8.3 to <11.2 minutes/bout; tertile 3: ≥11.2 minutes/bout), unbouted MVPA (shorter bouts [1-9 minutes]; tertile 1: <2.0 minutes/day; tertile 2: ≥2 to <7.8 minutes/day; tertile 3: ≥7.8 minutes/day), and bouted MVPA (longer bouts [≥10 minutes]; tertile 1: 0 minutes/day; tertile 2: >0 to <10.1 minutes/day; tertile 3: ≥10.1 minutes/day) with incident stroke risk.

Analyses were conducted using SAS software, version 9.4 (SAS Institute Inc). The significance threshold was 2-tailed *P* = .05.

## Results

### Participant Characteristics

Among 7607 participants, the mean (SD) age was 63.4 (8.5) years; 4145 participants (54.5%) were female, 3462 (45.5%) were male, 2407 (31.6%) were Black, and 5200 (68.4%) were White. A total of 2523 participants (33.2%) resided in the stroke belt, and 1638 (21.5%) resided in the stroke buckle. Overall, participants spent a mean (SD) of 190.5 (77.7) minutes per day in LIPA and 13.6 (17.9) minutes per day in MVPA, corresponding to a mean (SD) accelerometer wear time of 21.4% (8.5%) for LIPA and 1.5% (1.9%) for MVPA. Sedentary behavior accounted for a mean (SD) of 77.1% (9.4%) of wear time, equivalent to a mean (SD) of 12.3 (1.4) hours per 16-hour waking day. Additional participant characteristics stratified by tertiles of MVPA and sedentary time are shown in Table 1 and eTable 2 in the Supplement, respectively.

**Table 1.** Participant Characteristics by Tertiles of MVPA

Characteristic	Participants, No. (%)			*P* value
	MVPA tertile 1 (n = 2535)^a^	MVPA tertile 2 (n = 2533)^b^	MVPA tertile 3 (n = 2539)^c^	
**Baseline** ^d^				
Age, mean (SD), y	68.2 (7.7)	62.6 (7.7)	60.0 (7.8)	<.001
Sex				
Male	880 (34.7)	1160 (45.8)	1422 (56.0)	<.001
Female	1655 (65.3)	1373 (54.2)	1117 (44.0)	
Race				
Black	1061 (41.9)	790 (31.2)	556 (21.9)	<.001
White	1474 (58.1)	1743 (68.8)	1983 (78.1)	
Region of residence^e^				
Non–stroke belt or non–stroke buckle	1118 (44.1)	1097 (43.3)	1231 (48.5)	.001
Stroke buckle	550 (21.7)	542 (21.4)	546 (21.5)	
Stroke belt	867 (34.2)	894 (35.3)	762 (30.0)	
Educational level				
<High school	246 (9.7)	134 (5.3)	71 (2.8)	<.001
High school graduate	730 (28.8)	580 (22.9)	386 (15.2)	
Some college	740 (29.2)	704 (27.8)	574 (22.6)	
College graduate	816 (32.2)	1114 (44.0)	1506 (59.3)	
Current smoker	352 (13.9)	268 (10.6)	178 (7.0)	<.001
Alcohol consumption^f^				
None	1723 (68.0)	1396 (55.1)	1127 (44.4)	<.001
Moderate	715 (28.2)	1013 (40.0)	1267 (49.9)	
Heavy	96 (3.8)	124 (4.9)	145 (5.7)	
BMI, mean (SD)	29.7 (6.6)	28.8 (5.5)	27.1 (4.8)	<.001
Diabetes^g^	563 (22.2)	314 (12.4)	193 (7.6)	<.001
Hypertension^h^	1635 (64.5)	1259 (49.7)	939 (37.0)	<.001
Left ventricular hypertrophy	279 (11.0)	1646 (65.0)	140 (5.5)	<.001
Atrial fibrillation	220 (8.7)	144 (5.7)	107 (4.2)	<.001
History of CHD	408 (16.1)	296 (11.7)	226 (8.9)	<.001
**Accelerometer**				
Age at time of accelerometer testing, mean (SD), y	73.7 (8.8)	68.8 (7.9)	66.2 (8.0)	<.001
Season accelerometer worn^i^				
Summer	598 (23.6)	654 (25.8)	655 (25.8)	.16
Autumn	618 (24.4)	623 (24.6)	620 (24.4)	
Winter	612 (24.1)	557 (22.0)	571 (22.5)	
Spring	697 (27.5)	699 (27.6)	693 (27.3)	
Wear time, mean (SD), min/d	865.7 (105.6)	892.1 (101.0)	915.4 (93.5)	<.001
Valid wear days				
4-5	357 (14.1)	276 (10.9)	170 (6.7)	<.001
6-7	2178 (85.9)	2257 (89.1)	2369 (93.3)	
Sedentary time, mean (SD), min/d^j^	803.0 (63.8)	736.3 (64.3)	681.9 (73.4)	<.001
Sedentary bout duration, mean (SD), min/bout^k^	15.4 (12.1)	9.8 (3.3)	8.8 (2.9)	<.001
LIPA, mean (SD), min/d^l^	135.3 (62.7)	201.3 (64.1)	235.2 (69.7)	<.001
MVPA, mean (SD), min/d^m^	1.1 (1.1)	7.1 (3.1)	32.9 (19.4)	<.001
Bouted MVPA, mean (SD), min/d^n^	0	1.3 (2.6)	16.0 (17.5)	<.001

Abbreviations: BMI, body mass index (calculated as weight in kilograms divided by height in meters squared); CHD, coronary heart disease; LIPA, light-intensity physical activity; MVPA, moderate- to vigorous-intensity physical activity.

SI conversion factor: To convert fasting glucose from mg/dL to mmol/L, multiply by 0.0555.

^a^ Tertile 1 cutoff point was less than 2.7 minutes/day.

^b^ Tertile 2 cutoff point was at least 2.7 to less than 14.0 minutes/day.

^c^ Tertile 3 cutoff point was at least 14.0 minutes/day.

^d^ Demographic data, cardiovascular risk factors, chronic disease status, and medical history data were collected at the original baseline.

^e^ The stroke buckle includes the coastal plain region of Georgia, North Carolina, and South Carolina. The stroke belt includes the remainder of Georgia, North Carolina, and South Carolina plus Alabama, Arkansas, Louisiana, Mississippi, and Tennessee.

^f^ None indicates 0 drinks/week; moderate, >0 to 14 drinks/week for men and more than 0 to 7 drinks/week for women; and heavy, more than 14 drinks/week for men and >7 drinks/week for women.

^g^ Diabetes was defined as a fasting glucose of at least 126 mg/dL (or at least 200 mg/dL if the participant did not fast) or self-reported use of medications for glucose control.

^h^ Hypertension was determined as the mean of 2 blood pressure measures taken after 5 minutes of seated rest and defined as systolic blood pressure of at least 140 mm Hg, diastolic blood pressure of at least 90 mm Hg, or self-reported current use of antihypertensive medication.

^i^ Summer was defined as June 21 to September 20; autumn, September 21 to December 20; winter, December 21 to March 20; and spring, March 21 to June 20.

^j^ Sedentary time was defined as minutes in which the accelerometer registered less than 50 counts/minute. It was corrected for wear time and expressed as estimated minutes/day of sedentary time given a standardized 16 hours of accelerometer wear.

^k^ Sedentary bout was defined as consecutive minutes in which the accelerometer registered less than 50 counts/minute.

^l^ LIPA was defined as minutes in which the accelerometer registered 50 to 1064 counts/minute.

^m^ MVPA was defined as minutes in which the accelerometer registered at least 1065 counts/minute.

^n^ Bouted MVPA was defined as at least 10 minutes of consecutive accelerometer readings of at least 1065 counts/minute, allowing for 1- to 2-minute decreases below threshold.

### Physical Activity, Sedentary Behavior, and Incident Stroke

**Figure 1.**
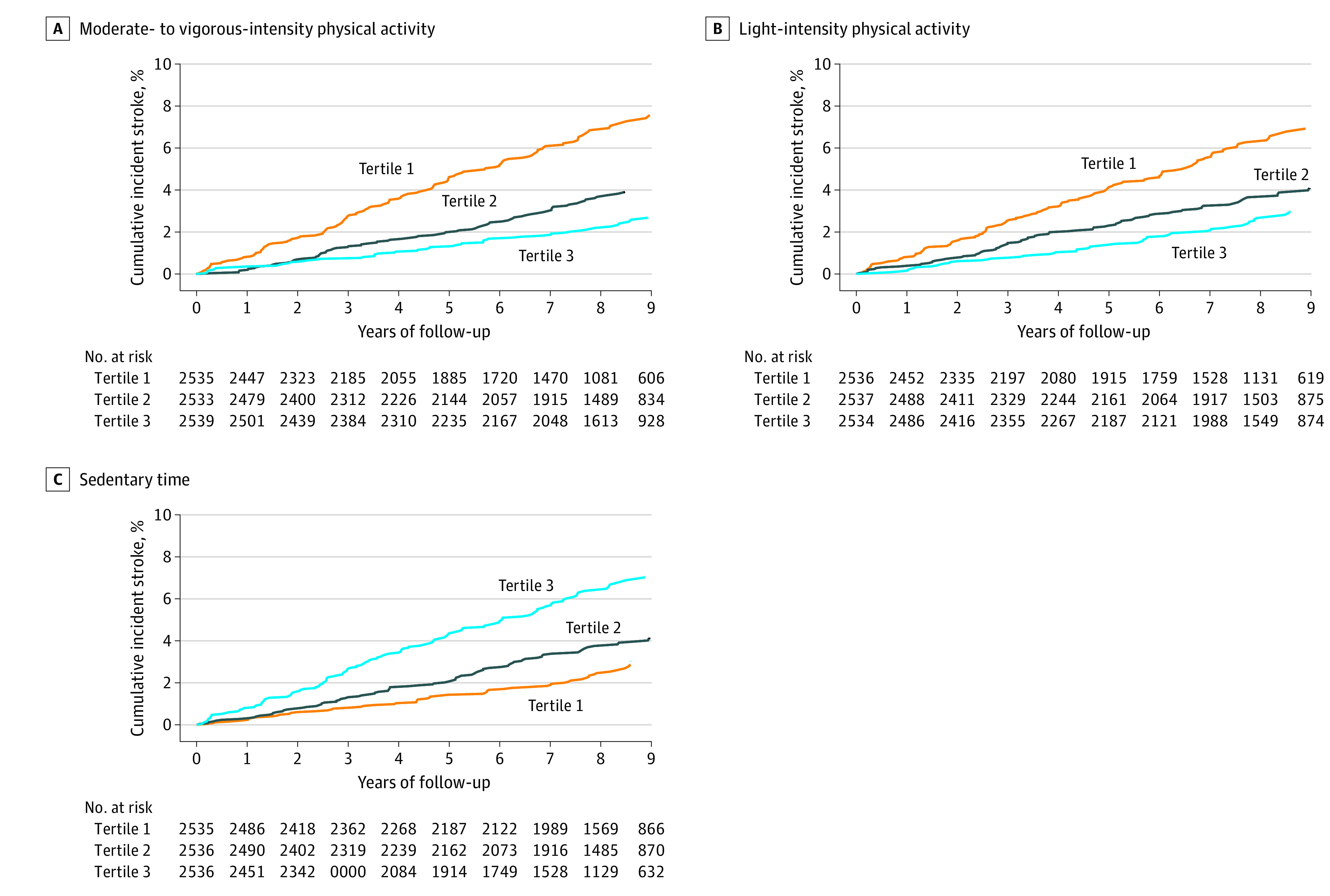
Cumulative Incident Stroke by Tertiles of Physical Activity and Sedentary Time A, Cutoff points were less than 2.7 minutes/day for tertile 1, at least 2.7 to less than 14.0 minutes/day for tertile 2, and at least 14.0 minutes/day for tertile 3. B, Cutoff points were less than 154.0 minutes/day for tertile 1, at least 154.0 to less than 220.4 minutes/day for tertile 2, and at least 220.4 minutes/day for tertile 3. C, Cutoff points were less than 11.8 hours/day for tertile 1, at least 11.8 to less than 13.0 hours/day for tertile 2, and at least 13.0 hours/day for tertile 3.

Over a mean (SD) follow-up of 7.4 (2.5) years, there were 286 incident stroke cases (244 [85.3%] ischemic and 42 [14.7%] hemorrhagic). When compared with the lowest tertile (tertile 1), LIPA and MVPA in the highest tertile (tertile 3) were associated with a significantly lower risk of incident stroke in model 1 (LIPA: 38% lower; HR, 0.62 [95% CI, 0.45-0.86; *P* = .003]; MVPA: 55% lower; HR, 0.45 [95% CI, 0.32-0.63; *P* < .001]) and model 2 (LIPA: 32% lower; HR, 0.68 [95% CI, 0.49-0.94; *P* = .02]; MVPA: 50% lower; HR, 0.50 [95% CI, 0.36-0.71; *P* < .001]) (Figure 1; Table 2). Greater sedentary time was associated with significantly higher risk of incident stroke in both model 1 (eg, tertile 3 vs tertile 1: 76% higher; HR, 1.76; 95% CI, 1.26-2.45; *P* < .001) and model 2 (eg, tertile 3 vs tertile 1: 60% higher; HR, 1.60; 95% CI, 1.15-2.23; *P* = .004). In model 3, mutual adjustment (ie, LIPA and sedentary time adjusted for MVPA; MVPA adjusted for sedentary time) yielded some attenuation in the point estimates, with results remaining statistically significant for MVPA (eg, tertile 3 vs tertile 1: 43% lower; HR, 0.57; 95% CI, 0.38-0.84; *P* = .004) and sedentary time (eg, tertile 3 vs tertile 1: 44% higher; HR, 1.44; 95% CI, 0.99-2.07; *P* = .04) but not for LIPA (eg, tertile 3 vs tertile 1: 26% lower; HR, 0.74; 95% CI, 0.53-1.04; *P* = .08). The amount of MVPA associated with a significant reduction in stroke risk was approximately 25 minutes per day (175 minutes/week). In secondary analyses with adjustment for risk factors that may have served as mediators, results for MVPA (eg, tertile 3 vs tertile 1: 42% lower; HR, 0.58; 95% CI, 0.39-0.86; *P* = .006) and sedentary time (eg, tertile 3 vs tertile 1: 41% higher; HR, 1.41; 95% CI, 0.97-2.05; *P* = .05) were also significant; however, LIPA was no longer associated with incident stroke risk (eg, tertile 3 vs tertile 1: 0.76; 95% CI, 0.54-1.08; *P* = .12) (eTable 3 in the Supplement). The associations of MVPA, LIPA, and sedentary time tertiles with incident stroke did not vary by age, sex, race, meeting vs not meeting physical activity guidelines, or body mass index category.

**Table 2.** Risk of Incident Stroke by Tertiles of Physical Activity and Sedentary Time

Model	Hazard ratio (95% CI)			*P* value for trend^a^
	Tertile 1	Tertile 2	Tertile 3	
**MVPA^b^**				
Stroke, No./total No.	147/2535	83/2533	56/2539	NA
Unadjusted	1 [Reference]	0.62 (0.47-0.82)	0.45 (0.33-0.62)	<.001
Model 1^c^	1 [Reference]	0.62 (0.47-0.83)	0.45 (0.32-0.63)	<.001
Model 2^d^	1 [Reference]	0.67 (0.50-0.89)	0.50 (0.36-0.71)	<.001
Model 3^e^	1 [Reference]	0.71 (0.52-0.96)	0.57 (0.38-0.84)	.004
**LIPA^f^**				
Stroke, No./total No.	137/2536	87/2537	62/2534	NA
Unadjusted	1 [Reference]	0.70 (0.53-0.92)	0.58 (0.42-0.79)	<.001
Model 1^c^	1 [Reference]	0.75 (0.57-0.99)	0.62 (0.45-0.86)	.003
Model 2^d^	1 [Reference]	0.80 (0.60-1.06)	0.68 (0.49-0.94)	.02
Model 3^e^	1 [Reference]	0.83 (0.63-1.11)	0.74 (0.53-1.04)	.08
**Sedentary time^g^**				
Stroke, No./total No.	59/2535	87/2536	140/2536	NA
Unadjusted	1 [Reference]	1.28 (0.91-1.78)	1.87 (1.35-2.58)	<.001
Model 1^c^	1 [Reference]	1.26 (0.90-1.77)	1.76 (1.26-2.45)	<.001
Model 2^d^	1 [Reference]	1.22 (0.87-1.70)	1.60 (1.15-2.23)	.004
Model 3^e^	1 [Reference]	1.13 (0.80-1.61)	1.44 (0.99-2.07)	.04

Abbreviations: LIPA, light-intensity physical activity; MVPA, moderate- to vigorous-intensity physical activity; NA, not applicable.

^a^ *P* value from linear trend test when tertiles were treated as an ordinal variable in the Cox proportional hazards regression model.

^b^ Cutoff points for MVPA were less than 2.7 minutes/day for tertile 1, at least 2.7 to less than 14.0 minutes/day for tertile 2, and at least 14.0 minutes/day for tertile 3.

^c^ Model 1 was adjusted for age, sex, race, region of residence (expressed as interaction with follow-up time), educational level, and season in which the accelerometer was worn.

^d^ Model 2 was adjusted for all covariates in model 1 plus current smoking, alcohol consumption, atrial fibrillation, left ventricular hypertrophy, and history of coronary heart disease.

^e^ Model 3 was adjusted for all covariates in model 2 plus MVPA (for sedentary time and LIPA) or sedentary time (for MVPA).

^f^ Cutoff points for LIPA were less than 154.0 minutes/day for tertile 1, at least 154.0 to less than 220.4 minutes/day for tertile 2, and at least 220.4 minutes/day for tertile 3.

^g^ Cutoff points for sedentary time were less than 11.8 hours/day for tertile 1, at least 11.8 to less than 13.0 hours/day for tertile 2, and at least 13.0 hours/day for tertile 3.

**Figure 2.**
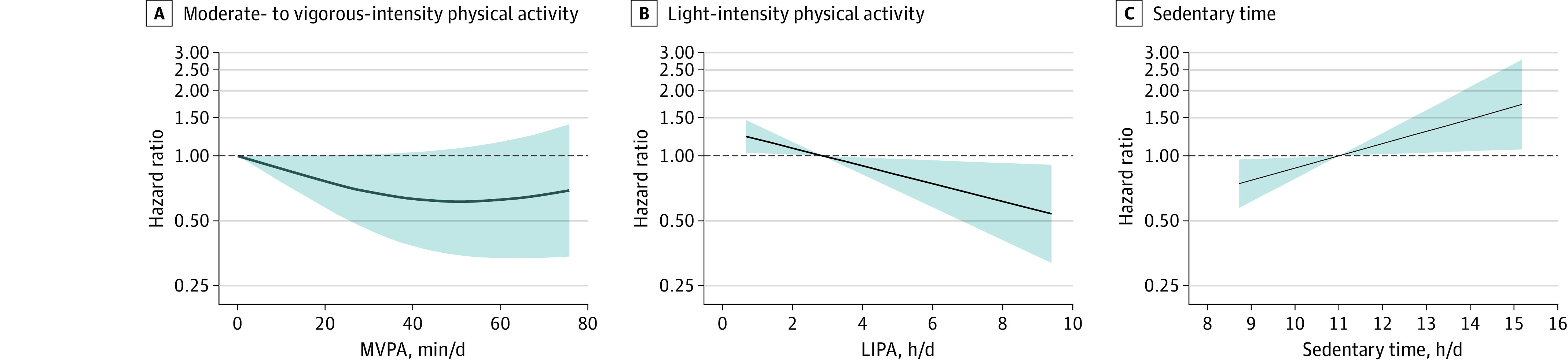
Dose-Response Association of Physical Activity and Sedentary Time With Risk of Incident Stroke Models were adjusted for age, race, sex, region of residence, educational level, season the accelerometer was worn, current smoking, alcohol use, atrial fibrillation, left ventricular hypertrophy, history of coronary heart disease, and either moderate- to vigorous-intensity physical activity for models testing sedentary time and light-intensity physical activity or sedentary time for models testing moderate- to vigorous-intensity physical activity. The dark blue lines represent hazard ratios, and shaded areas represent 95% CIs. A, Data were fitted using a nonlinear model (*P* = .15 for nonlinear association). Cubic polynomials were fitted with restrictions placed on the resulting curve to ensure a smooth appearance using 3 knots placed at the 5th, 20th, and 70th percentiles. The referent was the approximate median of the lowest tertile (0 minutes/day). B, Data were fitted using a linear model (*P* = .01). The referent was the approximate median of the lowest tertile (2.0 hours/day). C, Data were fitted using a linear model (*P* = .01). The referent was the approximate median of the lowest tertile (11.0 hours/16-hour day).

The dose-response associations with risk of incident stroke when MVPA, LIPA, and sedentary time were expressed continuously are shown in Figure 2. Sedentary time was associated with a significantly higher risk of incident stroke in a linear dose-response manner (HR per 1-hour/day increase in sedentary time: 1.14; 95% CI, 1.02-1.28; *P* = .02). Light-intensity physical activity was inversely associated with the risk of incident stroke in a linear dose-response manner (HR per 1-hour/day increase in LIPA: 0.86; 95% CI, 0.77-0.97; *P* = .02). No association was observed between MVPA and risk of incident stroke. Detailed data for Figure 2 are available in eTables 4, 5, and 6 in the Supplement.

### Bouted Physical Activity, Bouted Sedentary Behavior, and Incident Stroke

Mean sedentary bout duration was associated with a significantly higher risk of incident stroke in the unadjusted model (eg, tertile 3 vs tertile 1: HR, 1.85; 95% CI, 1.36-2.52; *P* < .001) and the multivariable-adjusted models (eg, tertile 3 vs tertile 1 in model 1: HR, 1.73; 95% CI, 1.26-2.37; *P* < .001) (Table 3; eFigure 2 in the Supplement). Longer bouts of sedentary time (≥17 minutes/bout) were associated with a 54% higher risk of stroke than shorter bouts (<8 minutes/bout). Unbouted MVPA (bouts of 1-9 minutes; eg, tertile 3 vs tertile 1 in model 1: HR, 0.48; 95% CI, 0.34-0.68; *P* < .001) and bouted MVPA (bouts of ≥10 minutes; eg, tertile 3 vs tertile 1 in model 1: HR, 0.64; 95% CI, 0.44-0.93; *P* = .009) were both associated with a significantly lower risk of incident stroke. After adjustment for sedentary time, the association between unbouted MVPA and incident stroke remained (eg, tertile 3 vs tertile 1 in model 3: HR, 0.62; 95% CI, 0.41-0.94; *P* = .02); however, bouted MVPA was no longer associated with incident stroke risk (eg, tertile 3 vs tertile 1 in model 3: HR, 0.78; 95% CI, 0.53-1.15; *P* = .17).

**Table 3.** Risk of Incident Stroke by Tertiles of Mean Sedentary Bout Duration and Bouted and Unbouted MVPA

Model	Hazard ratio (95% CI)			*P* value for trend^a^
	Tertile 1	Tertile 2	Tertile 3	
**Mean sedentary bout duration** ^b^				
Stroke, No./total No.	62/2535	86/2536	138/2536	NA
Unadjusted	1 [Reference]	1.23 (0.88-1.71)	1.85 (1.36-2.52)	<.001
Model 1^c^	1 [Reference]	1.22 (0.88-1.69)	1.73 (1.26-2.37)	<.001
Model 2^d^	1 [Reference]	1.20 (0.86-1.67)	1.64 (1.19-2.25)	.001
Model 3^e^	1 [Reference]	1.16 (0.83-1.62)	1.53 (1.10-2.12)	.008
**Unbouted MVPA** ^f^				
Stroke, No./total No.	144/2527	87/2542	55/2538	NA
Unadjusted	1 [Reference]	0.65 (0.50-0.85)	0.48 (0.35-0.67)	<.001
Model 1^c^	1 [Reference]	0.66 (0.50-0.87)	0.48 (0.34-0.68)	<.001
Model 2^d^	1 [Reference]	0.69 (0.52-0.91)	0.53 (0.38-0.76)	<.001
Model 3^e^	1 [Reference]	0.74 (0.55-1.00)	0.62 (0.41-0.94)	.02
**Bouted MVPA** ^g^				
Stroke, No./total No.	208/4762	44/1425	34/1420	NA
Unadjusted	1 [Reference]	0.73 (0.53-1.01)	0.59 (0.41-0.85)	.002
Model 1^c^	1 [Reference]	0.76 (0.54-1.05)	0.64 (0.44-0.93)	.009
Model 2^d^	1 [Reference]	0.81 (0.58-1.13)	0.69 (0.47-1.01)	.04
Model 3^e^	1 [Reference]	0.87 (0.62-1.22)	0.78 (0.53-1.15)	.17

Abbreviations: MVPA, moderate- to vigorous-intensity physical activity; NA, not applicable.

^a^ *P* value from linear trend test when tertiles were treated as an ordinal variable in the Cox proportional hazards regression model.

^b^ Cutoff points for mean sedentary bout duration were less than 8.3 minutes/bout for tertile 1, at least 8.3 to less than 11.2 minutes/bout for tertile 2, and at least 11.2 minutes/bout for tertile 3.

^c^ Model 1 was adjusted for age, sex, race, region of residence (expressed as interaction with follow-up time), educational level, and season in which the accelerometer was worn.

^d^ Model 2 was adjusted for all covariates in model 1 plus current smoking, alcohol consumption, atrial fibrillation, left ventricular hypertrophy, and history of coronary heart disease.

^e^ Model 3 was adjusted for all covariates in model 2 plus MVPA (for sedentary time and light-intensity physical activity) or sedentary time (for MVPA).

^f^ Cutoff points for unbouted MVPA were less than 2.0 minutes/day for tertile 1, at least 2.0 to less than 7.8 minutes/day for tertile 2, and at least 7.8 minutes/day for tertile 3.

^g^ Cutoff points for bouted MVPA were 0 minutes/day for tertile 1, more than 0 to less than 10.1 minutes/day for tertile 2, and at least 10.1 minutes/day for tertile 3.

## Discussion

In this cohort study of middle-aged and older Black and White adults in the US, as hypothesized, we observed that accelerometer-measured time spent in LIPA, MVPA, and sedentary behavior was significantly associated with the risk of incident stroke. Compared with participants in the lowest tertile, those in the highest tertile of total daily time in MVPA had a 43% lower risk of stroke. In contrast, sedentary time was associated with a 44% higher risk of stroke. These associations remained significant after adjusting for several potential confounders, including MVPA (for sedentary behavior), sedentary time (for MVPA), and mediators, suggesting independent associations with risk of stroke for these 2 components of the waking movement continuum.

Engaging in MVPA has been consistently associated with a reduced risk of stroke in several studies using self-reported physical activity measures.^3,19^ The significant benefit observed in those studies was associated with diverse physical activity measures, including vigorous physical activity,^20,21^ brisk walking,^22^ and moderate to higher levels of physical activity with regard to both intensity and duration.^23,24,25^ In the current study, the amount of MVPA associated with a significant reduction in stroke risk was approximately 25 minutes per day (175 minutes/week). This finding is consistent with existing physical activity guidelines (ie, ≥150 minutes/week of moderate physical activity), although further investigation to assess whether a lower level of MVPA is protective against stroke is warranted because our data suggest potential benefits at fewer than 25 minutes per day (Figure 2A).

The finding that, when expressed as a continuous variable, LIPA was associated with a lower risk of stroke was notable given that many middle-aged and older adults are not capable of or willing to engage in MVPA at the recommended levels. However, caution is advised because the amount of accumulated daily time spent in LIPA that was associated with reduced stroke risk in this cohort was 4 to 5 hours. This amount is not trivial and is likely only to be achieved with concerted effort. Our results were consistent with those of the Objectively Measured Physical Activity and Cardiovascular Health (OPACH) study,^26^ which reported that accelerometer-derived LIPA was associated with a reduced incidence of CHD and cardiovascular disease (CVD), including stroke, in older women. The amount of LIPA associated with a reduced risk of CHD and CVD events in the OPACH study (6.3 hours/day) was higher than that of our cohort.^26^ Yet, the OPACH study also reported a linear dose-dependent association of CHD and CVD events with increasing levels of LIPA. For each 1-hour increment in LIPA, there was a 14% reduction in CHD risk and an 8% reduction in CVD risk after adjustment for MVPA,^26^ which was similar to the 16% reduction in stroke risk per 1-hour increase in LIPA after adjustment for MVPA noted in the current study.

Time spent in sedentary behavior was of interest because most adults spend most of their awake time being physically inactive. Notably, participants in the highest tertile of sedentary time (≥13 hours/day) exhibited a 44% increase in risk of stroke compared with those in the lowest tertile (<11 hours/day). This association remained significant when adjusted for several covariates, including MVPA. Previous studies using self-reported television time as a proxy for sitting time have also reported a negative impact of sedentary behavior for the risk of stroke.^27,28,29,30^ In combination, the findings from our study and these previous studies^27,28,29,30^ suggest that the independent association of sedentary time with stroke risk is important for the implementation of comprehensive stroke risk reduction strategies.

To our knowledge, no other study has reported on the association of bout duration of physical activity or sedentary behavior with stroke risk. As expected, longer bouts of sedentary time (≥17 minutes/bout) were associated with a 54% higher risk of stroke than shorter bouts (<8 minutes/bout). The significant association was maintained after adjustment for several covariates, including MVPA, suggesting an independent association of sedentary bout duration with stroke risk. Cross-sectional studies^31,32^ have reported significant associations between measures of prolonged, uninterrupted sedentary bouts and cardiometabolic biomarkers. Experimental studies^33,34^ have revealed negative cardiometabolic outcomes after acute periods of prolonged, uninterrupted bouts of sedentary time compared with periodically interrupted bouts. Our findings provided additional data suggesting that the duration of sedentary bouts is an important consideration for cardiovascular health and future physical activity guidelines.

The biological mechanisms through which physical activity mediates the reduction in stroke risk are not precisely known, but it has been suggested to be multifactorial via modifiable risk factors,^35,36^ such as body fat, dyslipidemia, hypertension, and diabetes. Notably, the association of physical activity with stroke risk seems to be only partially explained by these risk factors because adjustment for them did not offset the protective benefits of physical activity.^35^ Our study also confirmed this finding. Thus, the protective benefit of physical activity goes beyond risk factor modification. Previous research^37,38,39,40^ found that higher levels of physical activity favorably act on carotid artery distensibility, nitric oxide availability, and endothelial dysfunction. These biological actions improve cardiovascular function via increased cerebral blood flow and brain volume, with a parallel delay in naturally decreasing cerebral tissue density.^41,42^ Physical activity may also provide anti-inflammatory benefits by reducing levels of C-reactive protein, which could mediate the impact of physical activity on stroke risk.^43,44^

### Strengths and Limitations

This study has several strengths. The study included a large population-based sample recruited from a well-characterized cohort of middle-aged and older Black and White adults living in the US. Participants had excellent adherence to the 7-day protocol, providing a large pool of high-quality accelerometer-derived data. The REGARDS study is a contemporary prospective cohort study with low rates of unavailability for follow-up and detailed phenotyping of participants based on an in-person baseline visit and frequent telephone contact. The REGARDS study also uses a rigorous stroke adjudication process. The measurement of risk factors before stroke events minimizes the likelihood of reverse causality for associations.

The study also has limitations. One limitation of using an accelerometer is that the types of physical activity and upper body movement are not captured. The accelerometer cannot distinguish between postures (such as sitting vs standing); thus, we relied on an intensity-only definition of sedentary behavior. Therefore, sedentary time might be overestimated because some standing may also have been included. Only 7 days of accelerometer data were collected; thus, the current study might have undersampled the exposures. A number of factors that were not captured in this study, such as orthopedic conditions and pain, could have had consequences for physical activity. Participant risk factors were collected at baseline, which was approximately 6 years before participants wore the accelerometer, and some of the risk factors (such as diabetes status) might have changed. It is also possible that physical activity may have changed during follow-up. Thus, residual confounding might exist from misclassification of participants with respect to important confounders. Because of the limited number of incident stroke events, we did not perform separate analyses to explore the associations of physical activity and sedentary time with risk of stroke subtypes (ie, ischemic and hemorrhagic).

## Conclusions

In this cohort study involving a geographically diverse, population-based sample of Black and White adults 45 years or older in the US, when analyzed across tertiles or as continuous variables, total time spent in both MVPA and sedentary time was significantly associated with the risk of incident stroke, even after mutual adjustment. When expressed continuously, total time spent in LIPA was also independently associated with the risk of incident stroke. Longer bouts of sedentary time were associated with a significantly higher risk of stroke, whereas longer bouts of LIPA were associated with a significantly lower risk of stroke. Unbouted MVPA was also significantly associated with the risk of stroke, suggesting that overall accumulation of MVPA is important. These results support recent clinical and public health guidelines encouraging people to move more and sit less to maintain cardiovascular health.

## Notes

Supplement. **eTable 1.** Characteristics of REGARDS Accelerometer Study Participants (N = 7607) by Accelerometer Wear Time **eTable 2.** Characteristics of REGARDS Accelerometer Study Participants (N = 7607) by Tertile of Sedentary Time **eTable 3.** Hazard Ratios for Risk of Incident Stroke by Moderate- to Vigorous-Intensity Physical Activity (MVPA), Light-Intensity Physical Activity (LIPA), and Sedentary Time Tertiles in the REGARDS Study (N = 7607) With Additional Adjustment for Potential Intermediary Pathway Variables (Body Mass Index, Diabetes, and Hypertension) **eTable 4.** Hazard Ratios for Incident Stroke by Moderate- to Vigorous-Intensity Physical Activity (MVPA) Levels in the REGARDS Study (N = 7607) **eTable 5.** Hazard Ratios for Incident Stroke by Light-Intensity Physical Activity (LIPA) Levels in the REGARDS Study (N = 7607) **eTable 6.** Hazard Ratios for Incident Stroke by Sedentary Time Levels in the REGARDS Study (N = 7607) **eFigure 1.** Inclusion Criteria for Examining the Association of Physical Activity and Sedentary Behavior With Incident Stroke Among Participants Enrolled in the REGARDS Study **eFigure 2.** Cumulative Incident Stroke by Tertiles of Sedentary Bout Duration and Bouted and Unbouted Moderate- to Vigorous-Intensity Physical Activity (MVPA)
