# Supplementary material for: Association of Accelerometer-Measured Sedentary Time and Physical Activity With Risk of Stroke Among US Adults
Source: JAMA Netw Open. 2022 Jun 3;5(6):e2215385. doi: 10.1001/jamanetworkopen.2022.15385 (PMC9166254; doi:10.1001/jamanetworkopen.2022.15385)
Supplement: Supplement. — eTable 1. Characteristics of REGARDS Accelerometer Study Participants (N = 7607) by Accelerometer Wear Time eTable 2. Characteristics of REGARDS Accelerometer Study Participants (N = 7607) by Tertile of Sedentary Time eTable 3. Hazard Ratios for Risk of Incident Stroke by Moderate- to Vigorous-Intensity Physical Activity (MVPA), Light-Intensity Physical Activity (LIPA), and Sedentary Time Tertiles in the REGARDS Study (N = 7607) With Additional Adjustment for Potential Intermediary Pathway Variables (Body Mass Index, Diabetes, and Hypertension) eTable 4. Hazard Ratios for Incident Stroke by Moderate- to Vigorous-Intensity Physical Activity (MVPA) Levels in the REGARDS Study (N = 7607) eTable 5. Hazard Ratios for Incident Stroke by Light-Intensity Physical Activity (LIPA) Levels in the REGARDS Study (N = 7607) eTable 6. Hazard Ratios for Incident Stroke by Sedentary Time Levels in the REGARDS Study (N = 7607) eFigure 1. Inclusion Criteria for Examining the Association of Physical Activity and Sedentary Behavior With Incident Stroke Among Participants Enrolled in the REGARDS Study eFigure 2. Cumulative Incident Stroke by Tertiles of Sedentary Bout Duration and Bouted and Unbouted Moderate- to Vigorous-Intensity Physical Activity (MVPA) [file jamanetwopen-e2215385-s001.pdf]

Supplementary Online Content

Hooker SP, Diaz KM, Blair SN, et al. Association of accelerometer-measured sedentary time and physical activity with risk of stroke among US adults. *JAMA Netw Open*. 2022;5(6):e2215385. doi:10.1001/jamanetworkopen.2022.15385

eTable 1. Characteristics of REGARDS Accelerometer Study Participants (N = 7607) by Accelerometer Wear Time

eTable 2. Characteristics of REGARDS Accelerometer Study Participants (N = 7607) by Tertile of Sedentary Time

eTable 3. Hazard Ratios for Risk of Incident Stroke by Moderate- to Vigorous-Intensity Physical Activity (MVPA), Light-Intensity Physical Activity (LIPA), and Sedentary Time Tertiles in the REGARDS Study (N = 7607) With Additional Adjustment for Potential Intermediary Pathway Variables (Body Mass Index, Diabetes, and Hypertension)

eTable 4. Hazard Ratios for Incident Stroke by Moderate- to Vigorous-Intensity Physical Activity (MVPA) Levels in the REGARDS Study (N = 7607)

eTable 5. Hazard Ratios for Incident Stroke by Light-Intensity Physical Activity (LIPA) Levels in the REGARDS Study (N = 7607)

eTable 6. Hazard Ratios for Incident Stroke by Sedentary Time Levels in the REGARDS Study (N = 7607)

eFigure 1. Inclusion Criteria for Examining the Association of Physical Activity and Sedentary Behavior With Incident Stroke Among Participants Enrolled in the REGARDS Study

eFigure 2. Cumulative Incident Stroke by Tertiles of Sedentary Bout Duration and Bouted and Unbouted Moderate- to Vigorous-Intensity Physical Activity (MVPA)

This supplementary material has been provided by the authors to give readers additional information about their work.

© 2022 Hooker SP et al. *JAMA Network Open*.

eTable 1. Characteristics of REGARDS Accelerometer Study Participants (N = 7607) by Accelerometer Wear Time

	Wear Time						
**Variable**	**10-12 h/d** *n=202*	**12-14 h/d** *n=2036*	**14-16 h/d** *n=3942*	**16-18 h/d** *n=1139*	**18-20 h/d** *n=151*	**≥20 h/d** *n=137*	**P-Value**
Baseline Data[a]							
Age	64.9 ± 8.8	64.7 ± 8.7	63.2 ± 8.4	62.0 ± 8.4	61.3 ± 8.2	61.7 ± 8.2	<0.001
Male, *n* (%)	82 (40.6)	843 (41.4)	1794 (45.5)	608 (53.4)	73 (48.3)	63 (46.0)	<0.001
Black race, *n* (%)	102 (50.5)	757 (37.2)	1001 (25.4)	354 (31.1)	77 (51.0)	71 (51.8)	0.098
Region of Residence,[b] *n* (%)							0.009
Non-belt/buckle	76 (37.6)	867 (42.6)	1821 (46.2)	552 (48.5)	73 (48.3)	58 (42.3)	
Stroke buckle	50 (24.8)	464 (22.8)	844 (21.4)	215 (18.9)	34 (22.5)	30 (21.9)	
Stroke belt	76 (37.6)	704 (34.6)	1277 (32.4)	372 (32.7)	44 (29.1)	49 (35.8)	
Education, *n* (%)							<0.001
Less than High School	27 (13.4)	169 (8.3)	193 (4.9)	42 (3.7)	6 (4.0)	13 (9.5)	
High School Graduate	70 (34.7)	538 (26.4)	812 (20.6)	213 (18.7)	37 (24.7)	26 (19.0)	
Some College	50 (24.8)	595 (29.2)	1013 (25.7)	279 (24.5)	43 (28.7)	39 (28.5)	
College graduate	55 (27.2)	735 (36.1)	1920 (48.7)	605 (53.1)	64 (42.7)	59 (43.1)	
Current Smoker, *n* (%)	32 (15.8)	271 (13.3)	355 (9.0)	101 (8.9)	20 (13.5)	18 (13.1)	0.001
Alcohol Drinking,[c] *n* (%)							0.025
None	142 (70.5)	1220 (59.9)	2093 (53.1)	616 (54.1)	99 (65.3)	78 (57.0)	
Moderate	52 (25.5)	709 (34.8)	1652 (41.9)	486 (42.7)	46 (30.6)	52 (37.8)	
Heavy	8 (4.0)	110 (5.4)	197 (5.0)	36 (3.2)	6 (4.1)	7 (5.2)	
Body Mass Index (kg/m^2)	29.7 ± 6.6	29.3 ± 5.8	28.3 ± 5.6	28.2 ± 5.6	28.9 ± 6.0	29.1 ± 5.5	<0.001
Diabetes,[d] *n* (%)	49 (24.1)	350 (17.2)	497 (12.6)	129 (11.3)	25 (16.4)	18 (13.4)	<0.001
Hypertension,[e] *n* (%)	130 (64.2)	1169 (57.4)	1896 (48.1)	500 (43.9)	73 (48.3)	68 (49.6)	<0.001
Dyslipidemia, *n* (%)	126 (62.6)	1226 (60.2)	2204 (55.9)	624 (54.8)	89 (58.9)	76 (55.6)	0.003
Left Ventricular Hypertrophy, *n* (%)	13 (6.6)	202 (9.9)	264 (6.7)	85 (7.5)	7 (4.7)	10 (7.4)	0.009

© 2022 Hooker SP et al. *JAMA Network Open*.

Atrial fibrillation, *n* (%)	11 (5.6)	145 (7.1)	240 (6.1)	57 (5.0)	7 (4.8)	11 (8.1)	0.161
History of CHD, *n* (%)	31 (15.2)	293 (14.4)	461 (11.7)	109 (9.6)	18 (12.2)	20 (14.8)	0.003
Accelerometer Data							
Age at time of accelerometer testing	71.5 ± 8.8	71.0 ± 8.8	69.4 ± 8.6	68.3 ± 8.6	67.6 ± 8.6	67.9 ± 8.3	<0.001
Season accelerometer worn,[f] *n* (%)							0.440
Summer	32 (15.8)	509 (25.0)	978 (24.8)	300 (26.3)	46 (30.5)	42 (30.7)	
Autumn	56 (27.7)	503 (24.7)	950 (24.1)	295 (25.9)	29 (19.2)	27 (19.7)	
Winter	58 (28.7)	503 (24.7)	930 (23.6)	202 (17.7)	28 (18.5)	29 (21.2)	
Spring	56 (27.7)	521 (25.6)	1084 (27.5)	342 (30.0)	48 (31.8)	39 (28.5)	
Wear Time (min/day)	690 ± 22	794 ± 32	897 ± 32	999 ± 30	1127 ± 36	1292 ± 52	<0.001
Valid wear days, *n* (%)							<0.001
4-5 days	106 (52.4)	358 (17.6)	252 (6.4)	62 (5.4)	13 (8.6)	11 (8.0)	
6-7 days	96 (47.5)	1676 (82.3)	3690 (93.6)	1077 (94.6)	138 (91.4)	126 (92.0)	
Sedentary Time[g] (min/day)	756 ± 74	751 ± 77	733 ± 82	729 ± 86	773 ± 103	805 ± 102	<0.001
Sedentary Bout Duration[h] (min/bout)	14.3 ± 14.3	12.2 ± 10.4	10.7 ± 6.9	10.4 ± 4.9	13.1 ± 8.4	14.4 ± 6.5	<0.001
LIPA[i] (min/day)	137 ± 69	163 ± 72	197 ± 74	220 ± 77	210 ± 91	214 ± 90	<0.001
MVPA[j] (min/day)	5.5 ± 9.9	8.2 ± 12.5	15.1 ± 18.7	19.3 ± 20.7	14.3 ± 21.4	15.5 ± 20.4	<0.001
MVPA Bouted[k] (min/day)	2.2 ± 6.6	3.1 ± 9	6.6 ± 13.2	8.4 ± 14.8	5 ± 12.9	6.3 ± 13.8	<0.001

Data presented as mean ± standard deviation or percent.

CHD, coronary heart disease; LIPA, light intensity physical activity; MVPA, moderate or vigorous intensity physical activity.

[a]Demographic data, cardiovascular risk factors, and chronic disease status/medical history data were collected at the original baseline.

[b]Stroke Buckle: coastal plain region of North Carolina, South Carolina, and Georgia; Stroke Belt: remainder of North Carolina, South Carolina, and Georgia, plus Alabama, Mississippi, Tennessee, Arkansas, and Louisiana.

[c]None: 0 drinks per week; Moderate: >0 to 14 drinks per week for males and >0 to 7 drinks per week for females; Heavy: >14 drinks per week for males and >7 drinks per week for females.
[d]Fasting glucose of 126 mg/dL or greater (or 200 mg/dL or greater if the participant failed to fast) or self-reported use of medications for glucose control.
[e]Determined as the mean of two blood pressures taken after 5-minutes of seated rest, and defined as a systolic blood pressure of ≥140 mmHg, diastolic blood pressure of ≥90 mmHg, or self-reported current use of anti-hypertensive medication.

© 2022 Hooker SP et al. *JAMA Network Open*.

[f]Summer: June 21-September 20; Autumn September 21-December 20; Winter: December 21-March 20; Spring: March 21-June 20.
[g]Minutes in which the accelerometer registered <50 counts per minute. Corrected for wear time and expressed as the estimated minutes/day of sedentary time given a standardized 16 hours of accelerometer wear (see Methods).
[h]Sedentary bout is defined as consecutive minutes in which the accelerometer registered <50 counts per min.
[i]Light intensity physical activity; minutes in which the accelerometer registered 50–1064 counts per minute.
[j]Minutes in which the accelerometer registered ≥1065 counts per minute.
[k]Defined as 10 or more minutes of consecutive accelerometer readings ≥1065 counts per minute allowing for 1-2-minute drops below threshold. The tertile cutoff points were <2.7, ≥2.7 to <14.0, and ≥14.0 min/day. The tertile cutoff points were <11.8, ≥11.8 to <13.0, and ≥13.0 h/day.

© 2022 Hooker SP et al. *JAMA Network Open*.

eTable 2. Characteristics of REGARDS Accelerometer Study Participants (N = 7607) by Tertile of Sedentary Time

**Variable**	**Tertile 1** *n=2535*	**Tertile 2** *n=2538*	**Tertile 3** *n=2534*	**P-Value**
Baseline Data[a]				
Age	64.9 ± 8.8	63.4 ± 8.4	62.3 ± 8.3	<0.001
Male, *n* (%)	1047 (41.3)	1141 (45)	1273 (50.2)	<0.001
Black race, *n* (%)	961 (37.9)	624 (24.6)	778 (30.7)	<0.001
Region of Residence,[b] *n* (%)				<0.001
Non-belt/buckle	1075 (42.4)	1146 (45.2)	1224 (48.3)	
Stroke buckle	581 (22.9)	537 (21.2)	520 (20.5)	
Stroke belt	877 (34.6)	852 (33.6)	793 (31.3)	
Education, *n* (%)				<0.001
Less than High School	218 (8.6)	132 (5.2)	104 (4.1)	
High School Graduate	664 (26.2)	532 (21)	499 (19.7)	
Some College	740 (29.2)	654 (25.8)	624 (24.6)	
College graduate	913 (36)	1217 (48)	1308 (51.6)	
Current Smoker, *n* (%)	332 (13.1)	238 (9.4)	226 (8.9)	<0.001
Alcohol Drinking,[c] *n* (%)				0.001
None	1521 (60)	1366 (53.9)	1359 (53.6)	
Moderate	877 (34.6)	1055 (41.6)	1065 (42)	
Heavy	137 (5.4)	114 (4.5)	112 (4.4)	
Body Mass Index (kg/m^2)	29.7 ± 5.5	28.3 ± 5.6	28.3 ± 5.6	<0.001
Diabetes,[d] *n* (%)	431 (17)	332 (13.1)	307 (12.1)	<0.001
Hypertension,[e] *n* (%)	1450 (57.2)	1235 (48.7)	1148 (45.3)	<0.001
Dyslipidemia, *n* (%)	1518 (59.9)	1422 (56.1)	1402 (55.3)	0.001
Left Ventricular Hypertrophy, *n* (%)	241 (9.5)	157 (6.2)	183 (7.2)	0.003
Atrial fibrillation, *n* (%)	175 (6.9)	157 (6.2)	139 (5.5)	0.048
History of CHD, *n* (%)	368 (14.5)	297 (11.7)	269 (10.6)	<0.001
Accelerometer Data				
Age at time of accelerometer testing	70.4 ± 8.8	69.6 ± 8.5	68.5 ± 8.5	<0.001
Season accelerometer worn,[f] *n* (%)				0.926
Summer	606 (23.9)	621 (24.5)	677 (26.7)	
Autumn	639 (25.2)	631 (24.9)	588 (23.2)	
Winter	634 (25)	596 (23.5)	520 (20.5)	
Spring	654 (25.8)	687 (27.1)	750 (29.6)	
Wear Time (min/day)	792 ± 45.1	885.3 ± 20.1	996.2 ± 89	0.001
Valid wear days, *n* (%)				<0.001
4-5 days	489 (19.3)	168 (6.6)	142 (5.6)	
6-7 days	2043 (80.6)	2365 (93.3)	2393 (94.4)	
Sedentary Time[g] (min/day)	750.2 ± 78.1	734.2 ± 81.6	736.3 ± 89.6	<0.001
Sedentary Bout Duration[h] (min/bout)	12.1 ± 11	10.7 ± 7.2	10.8 ± 5.7	<0.001
1LIPA[i] (min/day)	162.8 ± 72.6	194.5 ± 73.1	213.9 ± 78.4	<0.001
MVPA[j] (min/day)	8.8 ± 13.2	14.5 ± 18.6	17.9 ± 20.2	<0.001
MVPA Bouted[k] (min/day)	3.3 ± 8.8	6.4 ± 13.3	7.8 ± 14.2	<0.001
Data presented as mean ± standard deviation or percent.				

© 2022 Hooker SP et al. *JAMA Network Open*.

CHD, coronary heart disease; LIPA, light intensity physical activity; MVPA, moderate or vigorous intensity physical activity.
[a]Demographic data, cardiovascular risk factors, and chronic disease status/medical history data were collected at the original baseline.
[b]Stroke Buckle: coastal plain region of North Carolina, South Carolina, and Georgia; Stroke Belt: remainder of North Carolina, South Carolina, and Georgia, plus Alabama, Mississippi, Tennessee, Arkansas, and Louisiana.
[c]None: 0 drinks per week; Moderate: >0 to 14 drinks per week for males and >0 to 7 drinks per week for females; Heavy: >14 drinks per week for males and >7 drinks per week for females.<br>[d]Fasting glucose of 126 mg/dL or greater (or 200 mg/dL or greater if the participant failed to fast) or self-reported use of medications for glucose control.<br>[e]Determined as the mean of two blood pressures taken after 5-minutes of seated rest, and defined as a systolic blood pressure of ≥140 mmHg, diastolic blood pressure of ≥90 mmHg, or self-reported current use of anti-hypertensive medication.
[f]Summer: June 21-September 20; Autumn September 21-December 20; Winter: December 21-March 20; Spring: March 21-June 20.
[g]Minutes in which the accelerometer registered <50 counts per minute. Corrected for wear time and expressed as the estimated minutes/day of sedentary time given a standardized 16 hours of accelerometer wear (see Methods).
[h]Sedentary bout is defined as consecutive minutes in which the accelerometer registered <50 counts per min.
[i]Light intensity physical activity; minutes in which the accelerometer registered 50–1064 counts per minute.
[j]Minutes in which the accelerometer registered ≥1065 counts per minute.
[k]Defined as 10 or more minutes of consecutive accelerometer readings ≥1065 counts per minute allowing for 1-2-minute drops below threshold. The tertile cutoff points were <2.7, ≥2.7 to <14.0, and ≥14.0 min/day. The tertile cutoff points were <11.8, ≥11.8 to <13.0, and ≥13.0 h/day.

© 2022 Hooker SP et al. *JAMA Network Open*.

eTable 3. Hazard Ratios for Risk of Incident Stroke by Moderate- to Vigorous-Intensity Physical Activity (MVPA), Light-Intensity Physical Activity (LIPA), and Sedentary Time Tertiles in the REGARDS Study (N = 7607) With Additional Adjustment for Potential Intermediary Pathway Variables (Body Mass Index, Diabetes, and Hypertension)

	**Tertile 1**	**Tertile 2**	**Tertile 3**	**P-Trend**
MVPA	1.00 (ref)	0.72 (0.53-0.97)	0.58 (0.39-0.86)	0.006
LIPA	1.00 (ref)	0.84 (0.63-1.12)	0.76 (0.54-1.08)	0.115
Sedentary Time	1.00 (ref)	1.12 (0.79-1.60)	1.41 (0.97-2.05)	0.051
Data presented as hazard ratio (95% confidence interval).				
Models adjusted for age, sex, race, region of residence (expressed as interaction with follow-up time), education, season, current smoking, alcohol use, atrial fibrillation, left ventricular hypertrophy, history of coronary heart disease, MVPA (for sedentary time and LIPA) or sedentary time (for MVPA), body mass index, diabetes, and hypertension.				
The tertile cutoff points were <2.7, ≥2.7 to <14.0, and ≥14.0 min/day for MVPA; <154.0, ≥154.0 to <220.4, and ≥220.4 min/day for LIPA; and <11.8, ≥11.8 to <13.0, and ≥13.0 h/day for sedentary time				

© 2022 Hooker SP et al. *JAMA Network Open*.

eTable 4. Hazard Ratios for Incident Stroke by Moderate- to Vigorous-Intensity Physical Activity (MVPA) Levels in the REGARDS Study (N = 7607)

**MVPA** *(min/day)*	**HR (95% CI)**
0	1.00 (ref)
5	0.93 (0.87-1.00)
10	0.87 (0.76-1.00)
15	0.81 (0.66-1.01)
20	0.76 (0.58-1.01)
25	0.72 (0.51-1.01)
30	0.68 (0.45-1.02)
35	0.65 (0.41-1.03)
40	0.63 (0.38-1.04)
45	0.62 (0.36-1.06)
50	0.61 (0.35-1.08)
55	0.61 (0.34-1.12)
60	0.62 (0.34-1.16)
65	0.64 (0.34-1.22)
70	0.66 (0.34-1.29)
75	0.69 (0.34-1.38)
Model adjusted for age, race, sex, region of residence (expressed as interaction with follow-up time), education, season the accelerometer was worn, current smoking, alcohol use, atrial fibrillation, left ventricular hypertrophy, history of coronary heart disease, and MVPA	

© 2022 Hooker SP et al. *JAMA Network Open*.

eTable 5. Hazard Ratios for Incident Stroke by Light-Intensity Physical Activity (LIPA) Levels in the REGARDS Study (N = 7607)

**LIPA** *(h/day)*	**HR (95% CI)**
0.5	1.22 (1.03-1.46)
1.0	1.14 (1.02-1.28)
1.5	1.07 (1.01-1.13)
2.0	1.00 (ref)
2.5	0.93 (0.88-0.99)
3.0	0.87 (0.78-0.98)
3.5	0.82 (0.69-0.97)
4.0	0.76 (0.61-0.96)
4.5	0.71 (0.53-0.95)
5.0	0.67 (0.47-0.94)
5.5	0.62 (0.42-0.93)
6.0	0.58 (0.37-0.92)
6.5	0.54 (0.32-0.91)
Model adjusted for age, race, sex, region of residence (expressed as interaction with follow-up time), education, season the accelerometer was worn, current smoking, alcohol use, atrial fibrillation, left ventricular hypertrophy, history of coronary heart disease, and moderate to vigorous intensity physical activity (MVPA)	

© 2022 Hooker SP et al. *JAMA Network Open*.

eTable 6. Hazard Ratios for Incident Stroke by Sedentary Time Levels in the REGARDS Study (N = 7607)

**Sedentary Time** *(h/16-h day)*	**HR (95% CI)**
9.0	0.77 (0.61-0.97)
9.5	0.82 (0.69-0.98)
10.0	0.88 (0.78-0.98)
10.5	0.94 (0.89-0.99)
11.0	1.00 (ref)
11.5	1.07 (1.01-1.13)
12.0	1.14 (1.02-1.28)
12.5	1.24 (1.05-1.48)
13.0	1.30 (1.03-1.63)
13.5	1.39 (1.04-1.85)
14.0	1.48 (1.05-2.09)
14.5	1.58 (1.06-2.35)
15.0	1.69 (1.07-2.67)
Model adjusted for age, race, sex, region of residence (expressed as interaction with follow-up time), education, season the accelerometer was worn, current smoking, alcohol use, atrial fibrillation, left ventricular hypertrophy, history of coronary heart disease, and moderate to vigorous intensity physical activity (MVPA)	

© 2022 Hooker SP et al. *JAMA Network Open*.

eFigure 1. Inclusion Criteria for Examining the Association of Physical Activity and Sedentary Behavior With Incident Stroke Among Participants Enrolled in the REGARDS Study

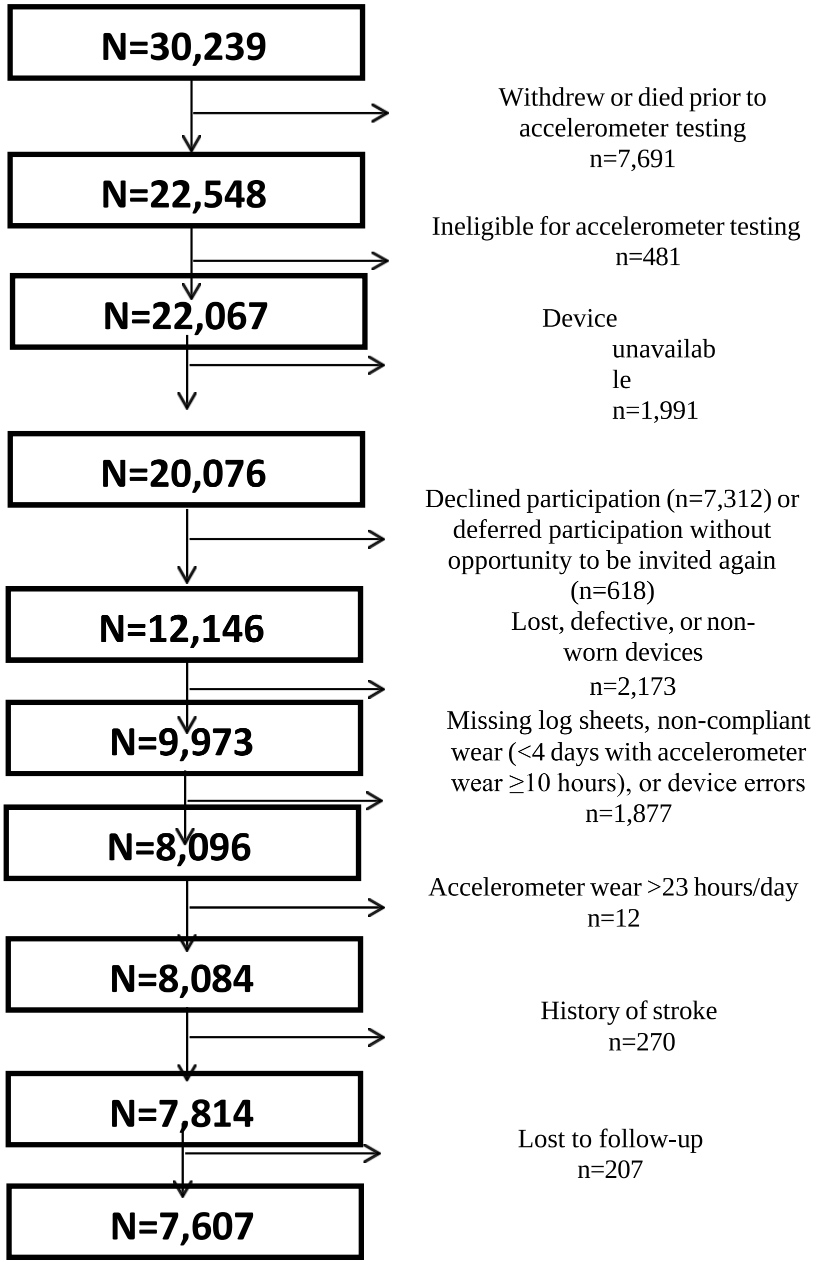

© 2022 Hooker SP et al. *JAMA Network Open*.

eFigure 2. Cumulative Incident Stroke by Tertiles of Sedentary Bout Duration and Bouted and Unbouted Moderate- to Vigorous-Intensity Physical Activity (MVPA)

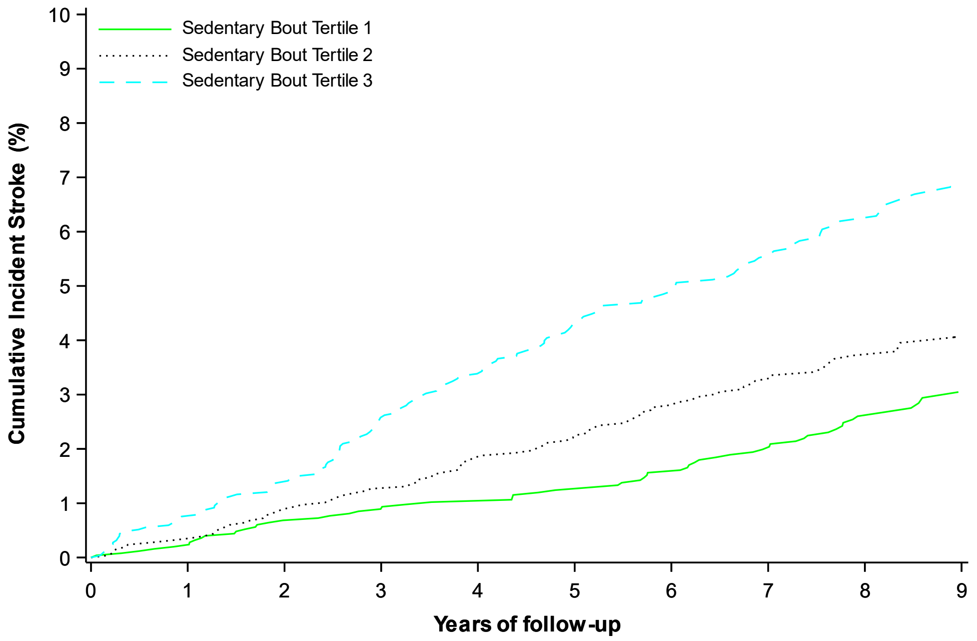

Participants at Risk (n)

**Tertile 1**	**2535**	**2487**	**2419**	**2359**	**2274**	**2192**	**2118**	**1970**	**1556**	**866**
**Tertile 2**	**2536**	**2483**	**2393**	**2306**	**2211**	**2127**	**2042**	**1884**	**1461**	**857**
**Tertile 3**	**2536**	**2457**	**2350**	**2216**	**2106**	**1944**	**1784**	**1579**	**1166**	**645**

© 2022 Hooker SP et al. *JAMA Network Open*.

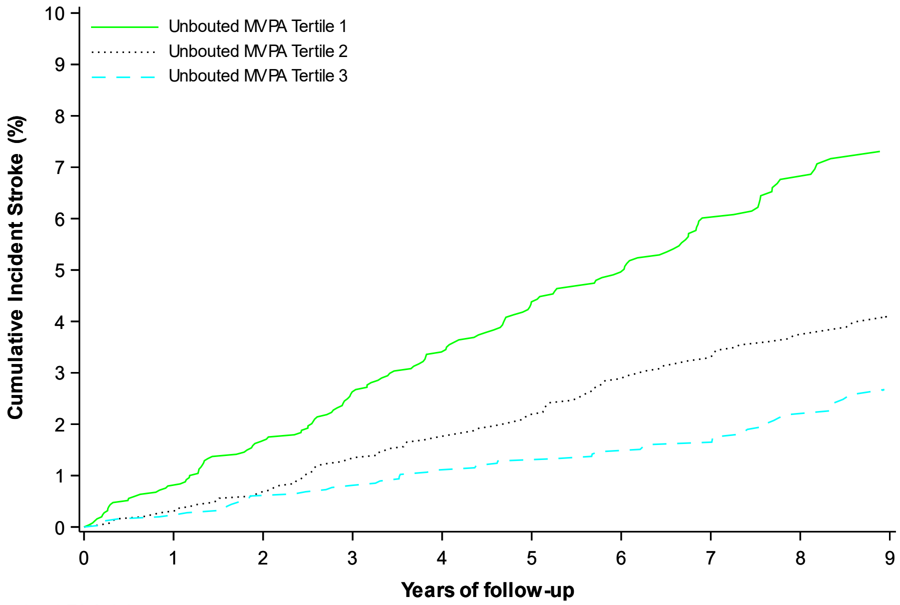

Participants at Risk (n)

**Tertile 1**	2527	2440	2320	2186	2067	1897	1745	1503	1099	617
**Tertile 2**	2542	2495	2413	2326	2236	2144	2039	1886	1500	866
**Tertile 3**	2538	2492	2429	2369	2288	2222	2160	2044	1583	885

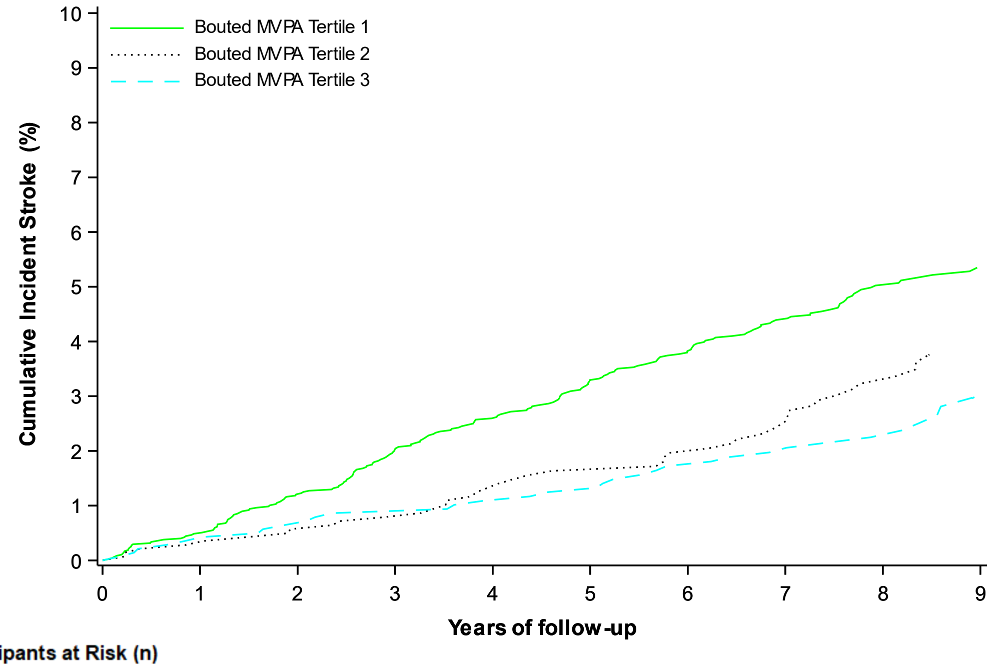

Participants at Risk (n)

**Tertile 1**	4762	4635	4437	4224	4022	3786	3542	3172	2401	1339
**Tertile 2**	1425	1397	1367	1333	1277	1225	1192	1113	869	493
**Tertile 3**	1420	1395	1358	1324	1292	1252	1210	1148	913	535

© 2022 Hooker SP et al. *JAMA Network Open*.
